# Ecosystem engineering of tundra heath by Arctic fox (*Vulpes lagopus*) is driven by nutrient additions

**DOI:** 10.1186/s13717-025-00646-5

**Published:** 2025-09-23

**Authors:** Liam Baron-Preston, James D. Roth, John H. Markham

**Affiliations:** Department of Biological Sciences, University of Manitoba, Winnipeg, MB Canada

**Keywords:** Tundra heath, Arctic fox, Ecosystem engineer, Nutrient limitation

## Abstract

**Background:**

Soil nutrient availability is a limiting factor for tundra productivity. Therefore, consumer-driven alteration of nutrient availability can have a large effect on tundra communities. Previous work has demonstrated that Arctic foxes (*Vulpes lagopus*) act as ecosystem engineers in tundra heath communities by altering plant composition and increasing plant biomass on their dens, which then increases snow depth. To test the ability of increased nutrients and deeper snow to cause the ecosystem effects observed on fox dens, we set up a nutrient addition and snow fencing experiment on tundra heath in Wapusk National Park, Canada.

**Results:**

Changes in experimental plots were mainly driven by fertilizer application, not snow depth. After 2 years, the fertilizer plots were invaded by a dune grass (*Elymus mollis*), which increased to 12% cover by the end of the experiment, which is typical of fox dens. After 4 years, total plant cover was 26% higher on the fertilizer plots than on the control plots. After 7 years of treatments, the plots receiving both fertilizer and snow fencing had the greatest shift in plant species composition, although they still lacked the tall willow shrubs typical of fox dens. Fertilized plots and dens had five times more arthropods than control plots. Most wildlife, except caribou (*Rangifer tarandus*), spent more time on fertilized plots in years when they were abundant, with Canada geese (*Branta canadensis*) being present 20 times longer in fertilizer plots. Collared lemmings (*Dicrostonyx richardsoni*) also preferred fertilized plots in the summer, but winter use was more pronounced on snow fenced and fertilized plots, where they produced 20 latrines per plot in a peak population year.

**Conclusions:**

These results demonstrate that the nutrient limitation in tundra vegetation makes tundra ecosystems vulnerable to changes in nutrient availability, with changes in plant abundance and composition leading to increased animal activity, that has the potential to create a positive feedback in ecosystem productivity.

**Supplementary Information:**

The online version contains supplementary material available at 10.1186/s13717-025-00646-5.

## Background

Arctic tundra ecosystems have long been characterized by extreme seasonality and low nutrient availability, which limit primary production and plant diversity. These limiting environmental conditions are now rapidly changing (Serreze et al. 2000), and large amounts of soil carbon and plant nutrients will potentially be made available to these ecosystems as soil warms and permafrost thaws (Keuper et al. 2012). Therefore, understanding the role that nutrients play in these systems is crucial.

The low productivity of tundra ecosystems is due to the climate’s direct and indirect effects. The short growing season directly limits plant growth. It also indirectly affects plant growth by limiting decomposition and mineralization of essential nutrients required for primary production, such that plants quickly exhaust soil resource pools in the growing season (Bliss et al. 1973; Sullivan et al. 2015; Keuper et al. 2012). The resulting buildup of organic matter further inhibits decomposition through poor soil aeration and creates a surface insulating layer, which decreases the depth to the permafrost layer (Grünberg et al. 2020). Low nutrient soils also become dominated by stress tolerant plants that produce tissues that are slow to decompose. The abundance of these plants is especially apparent in upland (heath) tundra communities that are dominated by prostrate shrubs, many of which are members of the Ericaceae, and form mycorrhizal relationships with fungi that are known for their ability to break down organic matter (Read et al. 2004). Therefore, there is potentially a positive feedback between plant tissue quality and nutrient availability (Orwin et al. 2010). Because tundra communities are nutrient limited, processes that increase nutrient availability can potentially have a large effect on entire tundra ecosystems. On the other hand, the buildup of a plant canopy in tundra heath in response to increased nutrient availability may be limited by snow depth, which protects plants in the winter (Gough et al. 2002).

Our previous work has described how Arctic foxes increase primary production and create atypical plant assemblages in tundra heath communities (Gharajehdaghipour et al. 2016; Fafard et al. 2019; Johnson-Bice et al. 2023). Arctic fox behaviour at their dens involves two processes: deposition of nutrients from prey remains and excreta and soil bioturbation from digging activity (Chesemore 1969; Smith et al. 1992). Burrows can be extensive, dens become littered with prey remains, and soils become more organic and acidic over time (Smith et al. 1992). This bioturbation may better disperse nutrients throughout the soil column (Smits et al. 1988) and benefit decomposers by aerating increasingly organic soils. With a significant energy cost for den construction, dens are reused and expanded year after year and may be abandoned for some years and then reoccupied (Gharajehdaghipour et al. 2016). Consequently, den sites in regions with permafrost can be hundreds of years old (Lang et al. 2022). Vegetation biomass is much greater on Arctic fox dens than in the surrounding tundra (Gharajehdaghipour et al. 2016), with dens being colonized by tall grasses and shrubs, not typically abundant on tundra heath (Fafard et al. 2019). The increased primary production is associated with significant greening of the tundra heath (Johnson-Bice et al. 2023). This may partly explain herbivorous wildlife’s greater use of den sites in the summer (Zhao et al. 2022). The shift in growth forms in tundra habitats also has the potential to alter insect communities (Richardson et al. 2002). Greater vegetation biomass modifies the physical structure of the environment. The increase in vegetation height and physical complexity traps blowing snow, increasing snow thickness on Arctic fox dens (Gharajehdaghipour and Roth 2018). Deeper snow insulates the ground from the colder air above and provides more water when it melts (Verstege 2016; Grünberg et al. 2020). Due to its effect on soil temperature, deeper snow allows for greater microbial activity during the winter and spring, potentially increasing N mineralization and nitrification in the soil (Schimel and Bennett 2004; Buckeridge et al. 2009). Deeper snow provides more insulation for the subnivean space, facilitating the establishment and survival of less cold tolerant plants more typical of forested habitats (Fafard et al. 2019), increasing plant diversity (Hooper et al. 2005). Deeper snow also increases habitat quality for small mammals. Gharajedaghipur and Roth (2018) found evidence that collared lemmings (*Dicrostonyx richardsoni*) preferentially use Arctic fox dens in the winter. Not only would lemming excreta further increase nutrient deposition (von Beckerath et al. 2022), but if the lemmings stayed to take advantage of higher quality forage in the spring, they could be an important food source for the foxes upon arrival. These cascading effects mean that foxes alter both community dynamics and ecosystem processes on their den sites, making them ecosystem engineers in tundra habitats (Jones et al. 2010).

Given the many differences that occur when foxes establish dens, we sought to separate the effects of two potential drivers of change, increased nutrients and increased snow depth, on tundra heath. Since tundra heaths are nutrient limited, we predicted that the strongest driver of change to communities would be due to nutrient addition, but that increased snow depth could provide protection from winter conditions, creating habitat for both plants and animals. We also predicted that an increased productivity from a shift in plant species composition would increase animal activity by providing a habitat for insects and a food source for wildlife. This experiment was also conducted to provide evidence that foxes modify their den habitats rather than select areas that are already different from the surrounding habitat (Johnson-Bice et al. 2023).

## Methods

### Experimental design and site description

In 2017, we set up a controlled experiment in Wapusk National Park, Canada, to simulate the effects of increased nutrients and snow depth on fox dens. We established 16 plots (10 m × 10 m) on a broad gravel ridge in *Dryas* tundra heath (Ponomarenko and Quirouette 2014) in an 8 × 2 grid with 100 m between plots. Each plot contained five permanent 1 m × 1 m quadrats used to track plant species’ cover over time (Supplementary Fig. 1). The plots were fertilized annually with 100 kg N ha^−1^ using urea and 28 kg P ha^−1^ using triple super phosphate and/or had snow fences (1.2 m high) erected on the plot’s windward (northwest) side. This application rate was partially based on an estimated mass of prey items brought to dens but also selected to conform with published fertilization experiments conducted in tundra vegetation. Each treatment combination was replicated four times such that four plots received fertilizer, four received snow fences, four received both treatments, and four received no treatments (controls). The treatments were arranged into two halves of a modified Latin square to control for spatial gradients (Supplementary Fig. 2). In 2022, eight Arctic fox dens were selected to compare the soil properties and insects with the experimental plots. In 2024, an additional 11 Arctic fox dens were selected for comparing plant species composition with the experimental plots. The chosen dens were randomly selected from dens within 8 km of the experimental plots that were also at least 15 years old, located in similar *Dryas* heath habitat and produced pups (been active) at least once since 2017. Compared to the treatment plots, the greater number of fox dens used in 2024 was chosen since fox dens can be quite variable in some of the plant species present.

### Abiotic factors

Snow depth in the experimental plots was measured in April from 2019 to 2024, except in 2020. In each plot, four measurements were taken, spaced 2.5 m from the centre of the NW to the SE edge of the plot, and then averaged. Soil moisture was measured using a theta probe on all plots on July 25, 2022, after five days of no precipitation. Nine measurements were taken in each plot, equally spaced across the plot. To keep disturbances to a minimum, soil samples were collected only once. At each experimental plot and fox den in July 2022, samples were collected 3.5 m from the centre of each plot and fox den in each of four directions (NW, NE, SE, SW). Each sampling took the top 10 cm of soil in a 5 cm equilateral triangle and the four samples were bulked. Soil inorganic N content (ammonium and nitrate) was determined using a microdiffusion assay (Khan et al. 2000). Phosphorus can be found in many different forms in soils. As such, the amount of soil P available to plants is difficult to estimate using a single assay method. Here, phytoavailable soil P was measured using the Murphy-Riley molybdate assay (Kalra and Maynard 1991), modified by using four different extractants to assess P available by different uptake strategies used by plants (DeLuca et al. 2015). These strategies include root interception (mimicked by CaCl_2_ solution), organic acid secretion (mimicked by citric acid), enzyme secretion (mimicked by phosphatase solution), and proton extrusion (mimicked by hydrochloric acid).

### Plant cover

The abundance of plant species was recorded for each experimental plot annually from 2017 (before the treatment application) to 2024 and for the 19 fox dens in 2024. The percent cover of each plant species was measured in each of the permanent quadrats in the experimental plots and their analogous locations at each fox den. For the experimental plots, the change in the presence or absence of each species was compared between the start of the experiment and 2024 to determine if species were invading or being lost from the quadrats. Cover values for each species were averaged per plot. Plants were classified into the following growth forms: prostrate shrubs, erect shrubs (> 25 cm in height), sedges and rushes, grasses, forbs, and mosses. The abundance of each functional group was calculated as the sum of the cover of each species of a functional group within a quadrat.

*Arctostaphylos alpina* is more common but indistinguishable from *Arctostaphylos rubra*. The two species were grouped as *A. alpina*. *Carex rupestris* is the most common *Carex* species on the experimental plots. In some years, *C. scirpodea* and *C. glacialis* may have been misidentified as *C. rupestris*. In years where these latter two species were identified, their mean cover on a plot never exceeded 1%. They were therefore grouped with *C. rupestris*. Two tall willows occur on fox dens, *Salix planifolia* and *Salix athabacensis*. Although they can be distinguished in the field, they grow intermixed, making the determination of their abundance difficult. They were therefore pooled together as *S. planifolia*. Mosses were pooled into a single category except for *Pleurozium schrebii* and *Dicranum elogatum* since most other species (e.g. *Ceratodon purpureus* and *Bryum* spp.) occurred as small mixed patches that, when dry, could not be distinguished. Although *Shepherdia canadensis* is a tall shrub in the southern part of its range, on the tundra it grows as a prostrate shrub and was classified as such. While it is common to group grasses and sedges into a common graminoid group, we kept them separate since the sedges on the tundra heath were all small (< 15 cm) and slow growing. In contrast, the grass species (*Elymus mollis and Hierochloe odorata*) were tall (> 30 cm) and fast growing.

### Animal abundance

Insects were sampled on the experimental plots and eight fox dens in 2022 by sweep netting. Three 1 m^2^ areas over the north, east, and south permanent quadrats were swept three times each using a 40 cm diameter net. Insects were pooled in each plot, identified to order, and then further organized into sub-order operational taxonomic units (OTUs) for better community resolution. The abundance of caribou (*Rangifer tarandus*), Canada geese (*Branta canadensis*), ptarmigan (*Lagopus lagopus*), and Arctic hares (*Lepus arcticus*) in experimental plots was estimated using fecal counts. In 2019, all feces of these species were removed from the permanent quadrats. Fecal counts started in subsequent years, counting and removing feces from the quadrats but not entirely from the plots each year. Trail cameras were also installed to record animal activity starting in July 2021. Cameras (Spypoint Force 20 or Reconyx PC900) were oriented to provide a field of view of the plot. The cameras were set to take a picture every minute when animal activity was detected. We counted the number of minutes animals spent on the plots from mid June to mid October (the snow free period) each year. The time on a plot was measured as the number of individuals within the plots multiplied by the number of minutes each animal was in the plot. In cases where groups of animals were in the plot, but the number of animals fluctuated between successive pictures, we assumed all the animals in the group were using the plot for the entire period. Technical problems with cameras (shifting out of the view of the plots, broken camera mounts, and camera failures) meant that we did not have a complete set of photos for each plot in a season. Therefore, cameras were used to confirm fecal count results and relate them to periods of animal occupancy in the plots. Lemming activity was first observed in 2021 (before this, we saw no evidence of lemmings on the plots) and recorded thereafter. Winter use of the plots was quantified by counting the number of lemming nests and latrines in the entire plot in early June, shortly after snowmelt. Nests and latrines were not removed after counting, but year old nests and latrines were easily distinguished from fresh ones. Burrows in the ground were enumerated to determine the spring and summer use of the plots.

### Statistical analyses

Statistical comparisons were performed in RStudio 4.4.0. Model fits were evaluated using DHARMa 4.6 (Hartig 2022). For the soil variables (collected in a single year with one value per plot), two-way generalized linear models (glms) were used to determine the effect of the snow fencing, fertilizer treatments and their interaction on the soil, plant and animal variables, using the nlme package version 3.1-164 (Pinheiro et al. 2023). Models were re-run with a single treatment if one of the treatments was not significant. For soil moisture data (with multiple samples per plot in the one year of sampling), the model included plot as a random variable. For data collected over multiple years (snow depth, cover of plant functional groups and animal activity), we used repeated measures mixed models, with treatments and years as fixed effects and plots as random effects. If the repeated measures model showed a significant effect of year, we then attempted to fit the data to generalized additive models (GAMs) using the ‘gam’ function in the mgcv package version 1.9-1 (Wood 2011), allowing each treatment to have its own thin spline smoother (Pedersen et al. 2019). When treatments were significantly different in the overall model, we compared differences in each year using the ‘difference_smooth’ command in the gratia package version 0.10.0 (Simpson 2024). To compare plant functional groups and insect abundance in the experimental plots to those on the Arctic fox dens, each plot was classified by the combination of the fencing and fertilizer treatment. For all models, data transformations or different data distributions were applied when appropriate. For the plant cover data, we compared models with a Gaussian distribution, with and without data transformation, to models with Poisson distributions. For the insect abundance data, we compared models with Gaussian and Poisson distributions. Since the fecal count data were highly variable among years, we also tested models with a negative binomial distribution and zero inflated Poisson models (‘family = ziP’). For the lemming burrow abundance analysis, there was no year effect, so the GAM was not run, and for the lemming latrine data, the GAM could not be fit. We therefore used a Tukey test from the repeated measures model to compare treatments within each year.

Plant and insect community composition in 2024 and 2022, respectively, among treatments and dens were assessed using non-metric multidimensional scaling (NMDS) ordinations and PERMANOVAs using the vegan package version 2.6.8 (Oksanen et al. 2024). Initial data assessment involved comparing correlations among treatments, using different similarity indices (Euclidean, Gower, Bray–Curtis, Hellinger, Chi squared, chord, Jaccard and Chao) on untransformed or log transformed data. The plant community NMDS was performed using the Bray dissimilarity index with a Wisconsin standardization of square root transformed data. The insect community NMDS was performed using a Bray dissimilarity index without data standardization (‘autotransform’ was set to FALSE in the ‘metaMDS’ function). One of the control plots had no insects captured using the standard collecting protocol. We therefore used a zero adjusted Bray index by adding a dummy species to each sample (Clarke et al. 2006). PERMNOVA was used to test for the difference between plot treatments and the dens, with ‘fdr’ *p* adjusted pairwise comparisons of insect OTU composition on dens with each experimental treatment combination. Plant and insect indicator species for the treatment plots and dens were determined using the indicspecies package version 1.7.15 (De Cáceres and Legendre 2009). We allowed the construction of groups of treatments to have indicator species by setting ‘duleg = FALSE’ in the ‘multipatt’ command.

## Results

### Abiotic factors

On plots with snow fences, snow depth increased to 112 ± 11 cm (mean ± standard deviation, averaged across all years), compared to 24 ± 14 cm on plots without (*p* < 0.0001 for the snow fence effect in a two-way repeated measures model). Snow depth was consistent among sampling years (*p* = 0.436). Plots accounted for 66% of the variation (Wald *p* = 0.026 for the plot effect from the REML variance component estimate). The fertilizer treatments had no effect on snow depth (*p* = 0.459) even though fertilizer increased the cover of tall grass in the fertilizer plots starting in 2021 (see below). The soil nutrient pools, measured after 5 years of fertilizer application, were not affected by the fencing treatment. Comparisons were therefore made between plots receiving fertilizer, or not, and fox dens. Soil inorganic nitrogen did not differ among treatment groups (*p* = 0.279 for a model on log transformed data comparing fertilized plots, non-fertilized plots and dens), with total inorganic N levels of 4.2 ± 1.9 µg g^−1^, suggesting any N fertilizer applied in previous years had been taken up or leached from the soil. Separating the total inorganic N levels into NH_4_^+^ and NO_x_^−^ fractions showed similar results (Supplementary Fig. 3). Phosphorus accessible by root interception (extracted by CaCl_2_) could only be detected in one of the eight non-fertilized plots (Table 1). The P levels extracted by CaCl_2_ on the fertilizer plots were similar to those found on dens. The other three P soil pools had significantly lower levels in the non-fertilizer plots, compared to fertilized plots and dens. Phosphorus accessible via organic acid release (extracted by citric acid) was three times lower on non-fertilizer plots than on the fertilizer plots and dens. Phosphorus accessible by phosphatase enzyme hydrolysis (extracted with an enzyme solution) was six times lower in non-fertilizer plots than in fertilized plots and dens. Phosphorus accessible by proton extrusion (extracted with HCl) was three times lower on non-fertilized plots than on fertilized plots and dens. The overall trends in soil N and P pools suggest the fertilizer treatments produced similar levels of nutrient availability to soil on fox dens. Soil moisture was not affected by the experimental treatments, but plots accounted for 75% of the variation in soil moisture (Wald *p* = 0.017), with mean soil moisture per plot ranging from 7 ± 3% to 43 ± 7% among the plots, with the driest plots occurring at the north end of the study site.

**Table 1:** Soil P levels (means ± standard deviation) using different extractants to assess different pools of soil P in experimental plots receiving no fertilizer, fertilizer, and soil from fox dens

Extractant	No fertilizer	Fertilizer	Den
CaCl_2_	0.4 ± 1.1	11.8 ± 18.1	15.6 ± 40.1
Citric acid	6.2 ± 4.3**A**	18.9 ± 6.4**B**	24.7 ± 16.9**B**
Phosphatase	4.1 ± 2.3**A**	23.4 ± 34.1**B**	7.4 ± 4.6**AB**
HCl	34.0 ± 14.2**A**	98.2 ± 35.9**B**	100.5 ± 64.7**B**

All values are in µg g^−1^. Letters following values are based on Tukey HSD tests. All analyses were performed on log transformed data. For the CaCl_2_ extraction, only fertilized plots and dens were compared since P was non detectible in all but one non-fertilized plot

### Plant cover

**Fig. 1:**
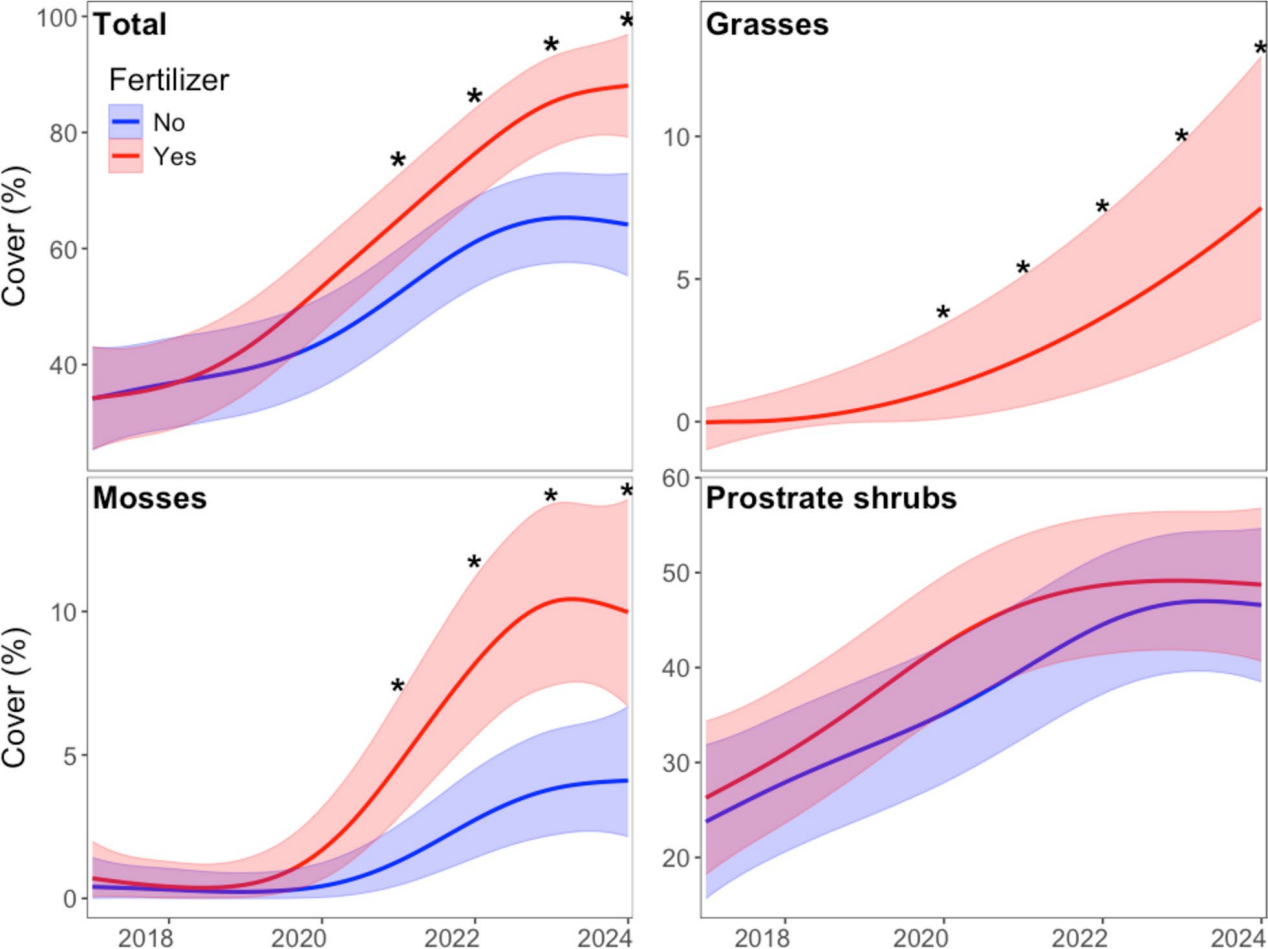
Predicted percent plant cover from generalized additive models. Shaded areas are 95% confidence intervals. An * indicates a significant difference between fence and fertilizer plot treatments. Models for grasses and mosses were run on square root transformed data, and the fitted values were back transformed for the figures. There were no grasses in the non-fertilized plots. Differences in grass cover between the fertilized and non-fertilized plots in Fig. 1 are based on the lower predicted confidence interval in the fertilized plots exceeding 0

Total plant cover, averaged over all treatments, was 34.7 ± 8.9% at the start of the experiment. Over the course of the study, plant cover increased in a non-linear manner on all plots (Fig. 1), but the increase was higher on fertilizer plots (*p* = 0.028 for the fertilizer effect and effective degree of freedom (*edf*)  = 3.407, *p* < 0.0001 for the fertilized plot smoother over years) than the non-fertilizer plots (*edf* = 3.496, *p* < 0.0001). Fertilizer plots started showing significantly higher cover than non-fertilized plots starting in 2021, i.e., four years after the start of the treatments. The increase in cover across all plots over time was driven by an increase in prostrate shrubs, mosses and sedges, and the increase in cover in the fertilized, compared to non-fertilized plots, was driven by an increased cover of grass and mosses. The tall grass, *Elymus mollis,* invaded six of the eight fertilizer plots. There was no grass on the non-fertilized plots (single culms were observed in 0 to 2 of the 40 non-fertilized plot quadrats in each year of the study, whereas in invaded plots, the grass formed patches of 100 to 1000 culms). Therefore, the non-fertilized plots were excluded from the GAM to account for changes in grass cover over time in the fertilized plots. A model using square root transformed data showed no smoother for year was needed (*edf* = 1.000, *p* < 0.0001), with grass cover increasing geometrically over time. Moss cover was initially quite low for the first three years of the study. It started to increase in 2021, with greater increases on fertilizer plots (*p* = 0.009 for the fertilizer effect and *edf* = 4.60, *p* < 0.0001 for the model with square root transformed data) than on the non-fertilized plots (*edf* = 3.94, *p* < 0.0001). Fertilization did not affect the abundance of prostrate shrubs (*p* = 0.396), but their cover almost doubled in the first six years of the experiment, levelling off in the last 3 years (*edf* = 3.30, *p* < 0.0001 for the fertilizer smoother and *edf* = 3.465, *p* < 0.0001 for the non-fertilizer smoother). The cover of forbs (not shown) remained low throughout the experiment (less than 3% cover) with no effect of fertilizer (*p* = 0.590). Although the smoother terms for forb cover over time were significant (*edf* = 5.554, *p* = 0.0002 for the fertilizer smoother and *edf* = 5.790, *p* < 0.0001 for the non-fertilizer smoother), there was no net increase in forb cover over time. Similarly, the cover of sedges (not shown) was not affected by fertilizer (*p* = 0.895). Snow fencing did not affect the cover of any plant group.

**Fig. 2:**
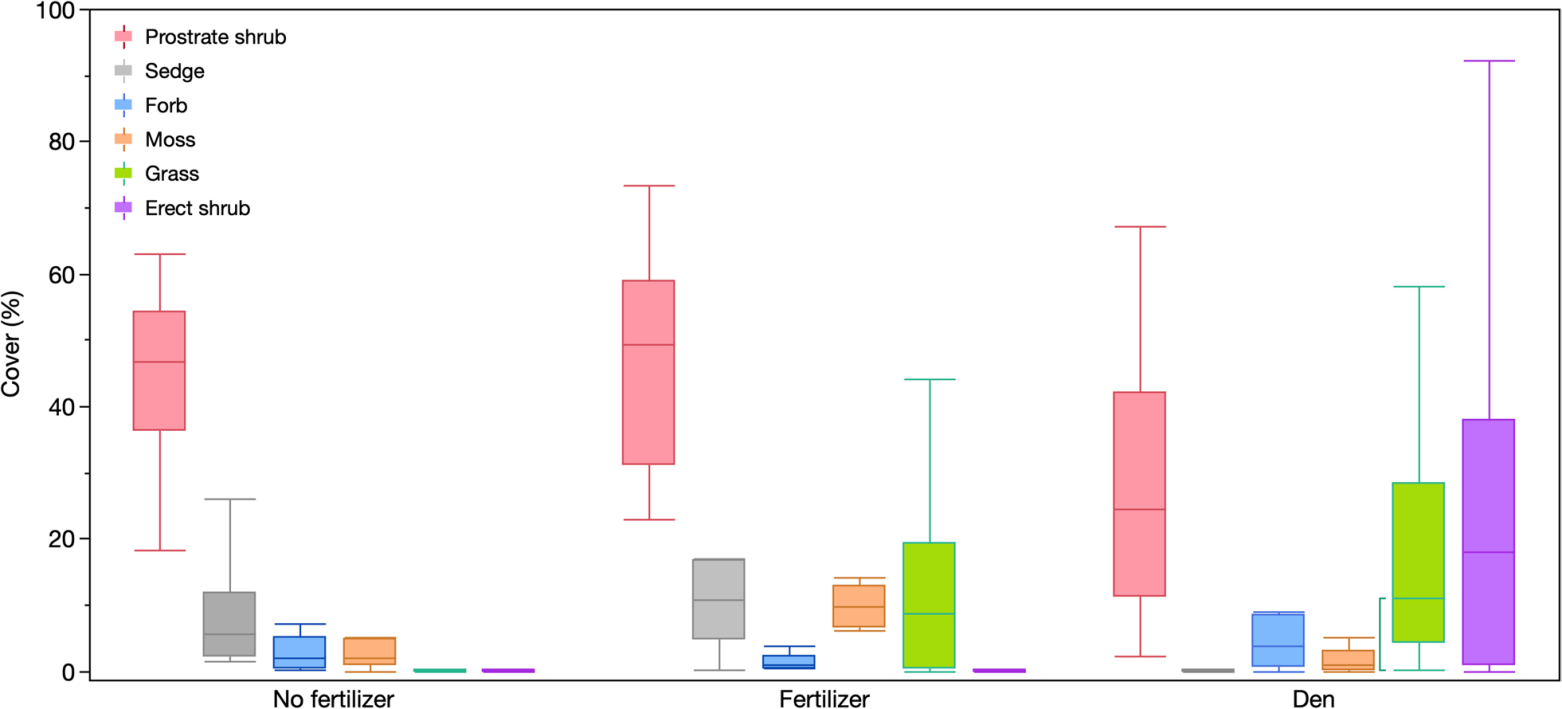
Abundance of plant groups on plots receiving fertilizer or not for 7 years (*n* = 8) and fox dens in the region (*n* = 19)

Although the experiment was set up to mimic the vegetation composition found on fox dens, the treatments did not result in a comparable abundance of all plant groups (Fig. 2). Erect shrubs are a major component of fox dens, having more than 10% cover on 13 of the 19 dens, but were only found on three of the experimental plots and never exceeded 0.2% cover. Sedge cover was higher on the experimental plots, regardless of fertilizer treatment, than on fox dens (*p* < 0.0001 for an ANOVA on square root transformed data comparing fertilized plots, non-fertilized plots and dens). Moss cover was higher on the fertilizer plots than the dens (*p* = 0.001 for an ANOVA on square root transformed data). The grass cover found on dens (almost exclusively *E. mollis*) did not differ from the fertilizer plots (*p* = 0.396, for an ANOVA with the non-fertilized plots excluded because they had no grasses). There was no difference among fertilized plots, non-fertilized plots and dens in the cover of prostrate shrubs (*p* = 0.107) or forbs (*p* = 0.076 for square root transformed data).

**Fig. 3:**
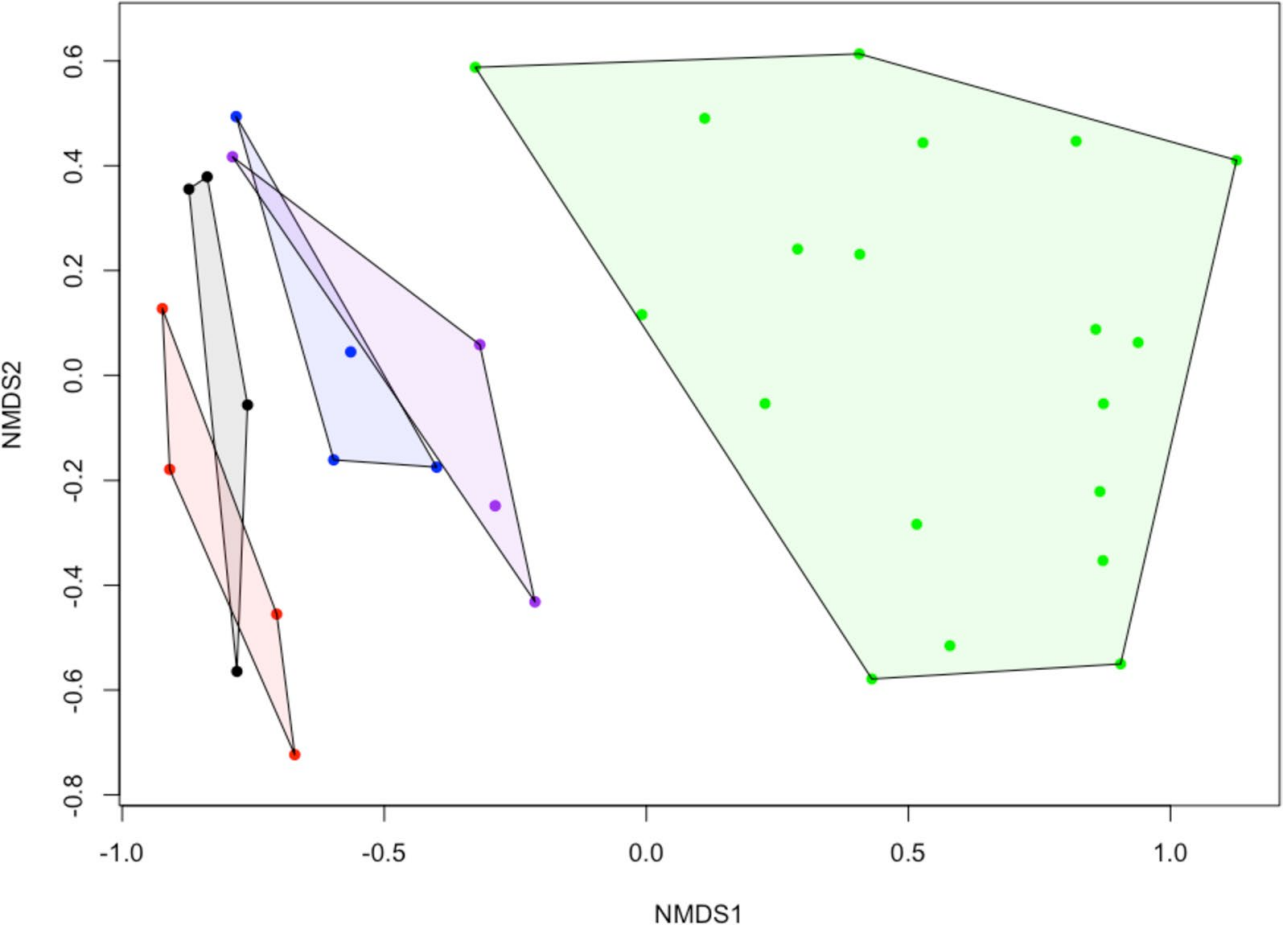
NMDS of plant species composition of treatment plots and fox dens in 2024 based on a Bray–Curtis dissimilarity index on log transformed and Wisconsin standardized data (stress = 0.161). Colour codes: Black = Control; Red = Fence; Blue = Fertilizer; Purple = Fence + fertilizer; Green = Dens

The largest change in the presence and absence of species occurred in the plots receiving both snow fencing and fertilizer (Supplementary Table 1). In these plots, the species showing the most change were: the prostrate shrub *Rhododendron lapponicum*, being lost in 55% of the quadrats; the forb *Toefeldia pusilla,* being lost from 40% of the quadrats; the grass *Elymus mollis*, invading 45% of the quadrats; and the forb *Chaemerion angustifolia*, invading 30% of the quadrats. An ordination of species composition also suggests that the treatments have yet to mimic dens in plant species composition (Fig. 3). During the first three years of the study, the treatment plots completely overlapped (not shown). By 2024, plots receiving fertilizer and snow fences had shifted closer to dens than any other treatment, followed by plots receiving only fertilizer. Dens were much more variable in species composition than treatment plots. Given this high variability, dens could not be compared to treatment plots using PERMANOVA. Indicator species analysis showed three species were indicators of dens alone (*Salix planifolia*, *Rubus acaulis*, and *Pyrola grandiflora*), all of which were never found on any quadrats of the experimental treatment plots. However, in 2024 we found single *S. planifolia* seedlings on three of the eight plots receiving fertilizer (but outside the permanent quadrats), suggesting that these tall shrubs take longer than grasses to respond to a nutrient addition. *Elymus mollis* and *Chaemerion angustifolia* were indicators of dens and plots receiving fertilizer. *Tofeldia pusila* and *Saxifraga azoides* were indicators of the experimental plots that did not receive fertilizer. There were also a group of species that were indicators of non-den sites: *Dryas integrifolia*, *Carex rupestris*, *Vaccinium uligonosum* and *Rhododendron lapponicum*, the latter two species never being found on dens even though they are common throughout the beach ridge habitats where foxes den.

### Animal abundance

**Fig. 4:**
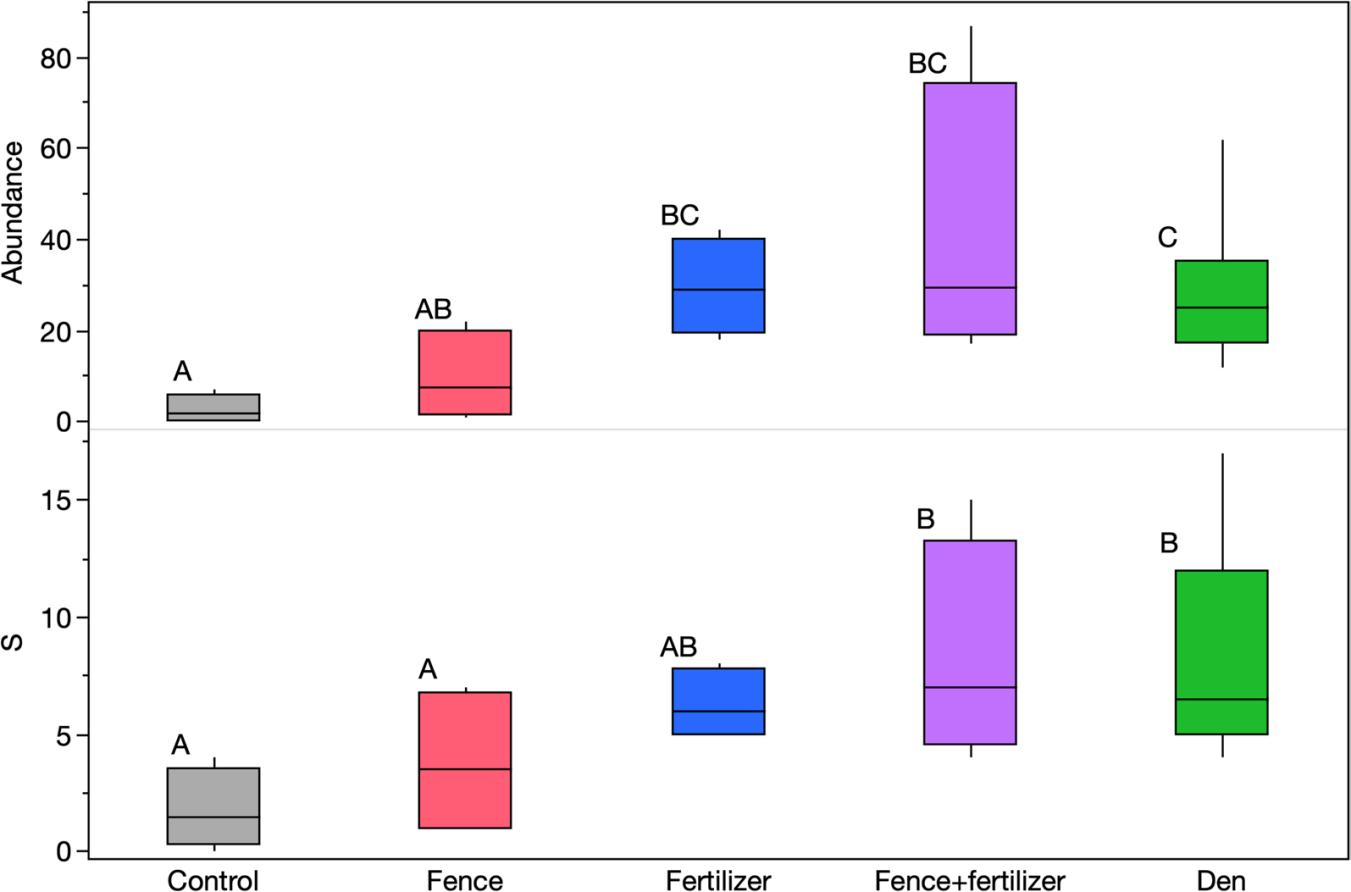
Total arthropod abundance and richness (S) of OTUs collected by net sweeps at experimental plots and fox dens in July 2022. Boxes encompass the 25th to 75th quantiles and the whiskers extending to the outermost data points. Letters indicate differences between treatments according to a Tukey’s test. Abundance data were log transformed for the glm fit

Net sweeps conducted on the experimental plots and fox dens in 2022 found arthropods from six insect orders (Diptera, Hemiptera, Hymenoptera, Lepidoptera, Orthoptera and Trichoptera). In addition, three Araneae were collected on one den site. Arthropods were organized into 39 OTUs (Supplementary Table 2). Over half (23) of the insect OTUs were dipterans, most of which were found on either fox dens (18) or plots with both snow fencing and fertilizer (13). No arthropods were found in one of the control plots. We found significantly higher arthropod abundance on dens and experimental plots that received fertilizer than in control plots (Fig. 4, *p* = 0.0002 for a glm comparing experimental plot treatment combinations and dens using log transformed data with a Gaussian distribution). Plots with only fencing were statistically similar to controls and plots that received fertilizer but had a lower abundance than plots with both snow fencing and fertilizer, as well as fox dens. OTU richness showed a similar pattern, with fox dens and experimental plots receiving snow fencing and fertilizer having a higher richness than control plots, with no significant differences between snow fence only and control plots (*p* = 0.006 for a glm on log transformed data with a Gaussian distribution).

**Fig. 5:**
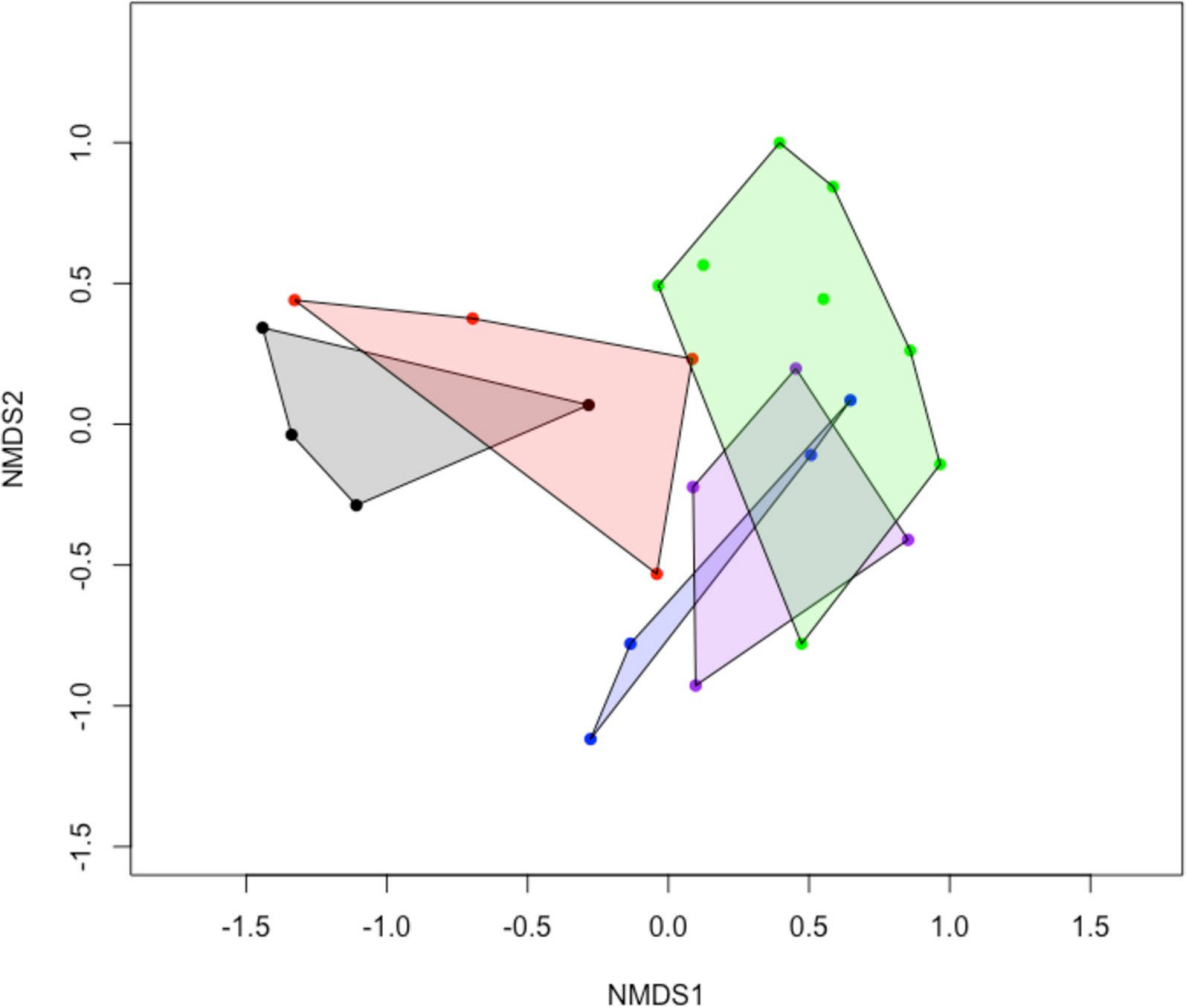
Zero adjusted NMDS of insect OTU abundance (stress = 0.156). Colour codes: Black = Control; Red = Fence; Blue = Fertilizer; Purple = Fence + fertilizer; Green = Dens

An ordination of the insect communities showed dens overlapped with plots receiving fertilizer and they were distinct in their composition from control and snow fenced plots (Fig. 5). Contrasts between plots and treatments from a PERMANOVA model showed that control and fertilizer plots were different from the dens. Indicator species analysis found that none of the arthropod OTUs were indicators of individual treatments or dens. There were three indicator OTUs. One dipteran from the superfamily Muscoidea was an indicator of dens and plots receiving fertilizer and was found on 82% of these plots. Another dipteran from the suborder Nematocera was an indicator of all sample types except control plots and was found in 90% of samples that did not come from control plots. One hemipteran from the family Delphacidae was an indicator of plots that received fertilizer, with or without snow fencing, and was found on 71% of these plots.

**Fig. 6:**
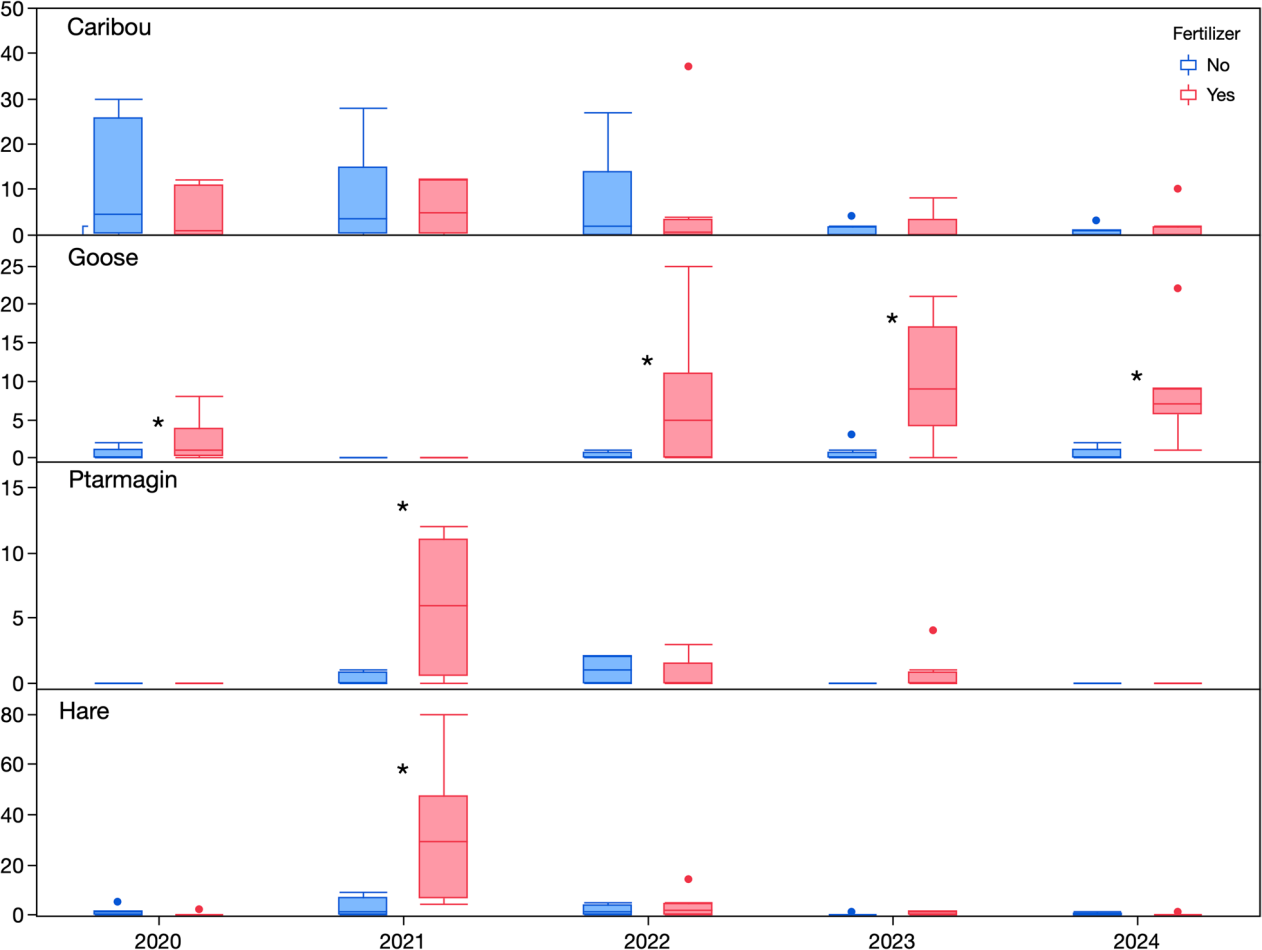
Wildlife fecal counts. *Indicates significant differences between fertilized and non-fertilized plots according to GAM models with separate smoothers for each treatment over time and negative binomial (caribou) or zero inflated Poisson (goose, hare and ptarmigan) distributions

Fecal counts showed all species of wildlife except caribou preferred fertilized plots (Fig. 6). Caribou fecal counts declined throughout the study. The decline corresponded to caribou spending an average of 21 min on a plot in 2020 to 4 min in 2024. There were significantly more goose feces on fertilizer plots in all years except in 2021, when goose feces were completely absent. This absence of goose feces in 2021 was confirmed by trail cameras showing only 21 and 27 min of goose activity in two plots (both fertilizer plus snow fence plots) compared to an average of 389 min in fertilized plots. Generally, when trail cameras captured geese, the geese spent some time feeding. In our vegetation surveys, there was clear evidence of feeding on *Elymus mollis*. In 2021, the only year that ptarmigan and hare feces were abundant, they were significantly more abundant on fertilizer plots.

**Fig. 7:**
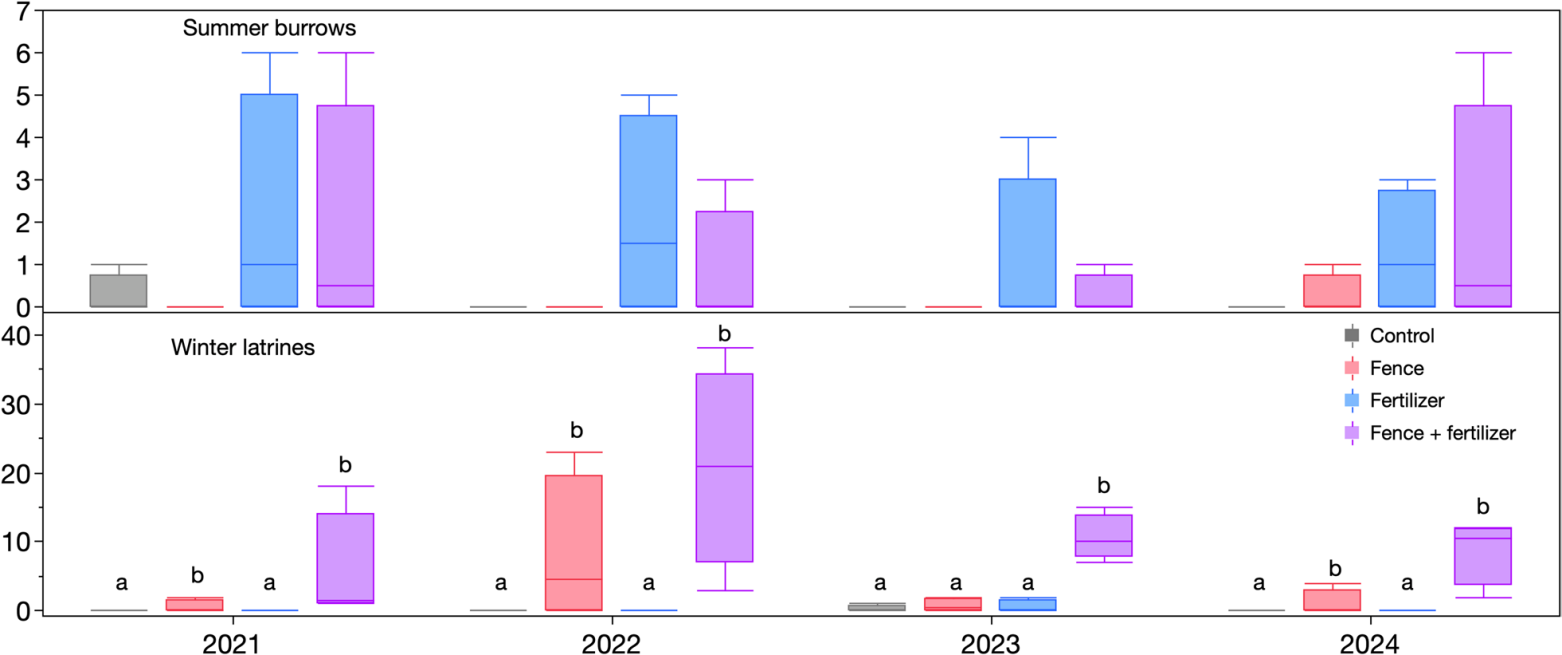
Lemming activity as indicated by summer burrows and winter latrines. Burrows were more common on fertilized plots regardless of snow fencing or year, according to a GAM model. Both snow fencing and fertilizer increased the number of latrines. Letters over winter latrine bars indicate differences between treatments within a year from a repeated measures model

Summer lemming activity, measured as the number of burrows in the ground, was higher on plots receiving fertilizer, with burrows only being found two out of 32 times (on eight plots over 4 years) on non-fertilized plots, compared to 13 times on fertilized plots (Fig. 7). A repeated measures model with a Poisson distribution showed a significant fertilizer effect (*p* = 0.005) but no effect of time or snow fencing. Winter lemming activity, measured as the number of latrines, was affected by snow fencing (*p* < 0.0001 for a repeated measures glmm with a Poisson distribution), fertilizer (*p* < 0.008) and year (*p* < 0.0001), but no interaction between snow fencing and fertilizer. In all but 2023, snow fencing and snow fencing in combination with fertilizer increased winter activity on the plots. In 2022, there was a large increase in lemming latrines, with the fenced and fertilized plots having an average of 20.1 latrines, which were sustained in 2023 and 2024. This increase was associated with patches of dead *Dryas integrifolia* and *Rhododendron lapponicum* occurring in these plots due to lemmings feeding on the stems of plants (Supplementary Fig. 4).

## Discussion

Overall, these results provide strong evidence that tundra heath communities are nutrient limited and that Arctic foxes engineer their den sites mainly through nutrient deposition. Although foxes create substantial soil disturbances, which are often associated with ecosystem engineering (Berke 2010), we did not find that fox dens had plants associated with disturbance that were not also found on our treatment plots. Indeed, fireweed (*Chaemerion angustifolia*), which is typically associated with disturbance, was found on dens and invaded the fertilized plots. This colonization of undisturbed fertilizer plots likely occurred since the heath habitat contains bare ground that small seeded weedy plants can easily invade. The changes in vegetation brought about by increased nutrient availability resulted in increased insect and vertebrate abundance, showing that nutrient enrichment results in a modification to the entire tundra food web. It has been suggested that biotic resistance appears to be more important than abiotic factors as an inhibitor of invasion in subarctic plant communities (Milbau et al. 2013). Our study suggests that greater nutrient availability could decrease biotic resistance by reducing abiotic limits. With more nutrients available to plants, competition between plant species would shift from below-ground nutrient acquisition from fungal partners to above-ground competition for light. Any selective pressure for plants with highly specialized mycorrhizal associations would cease, and previously bare ground may be more suitable for colonization. The loss of the ericaceous species *Rhodendron lapponicum* and *Vaccinium uligonsum* on the fertilized plots indicates that these common plants on heaths are at a competitive disadvantage. This loss supports the concept that ecosystem engineering can shift a community along a stress gradient where competition becomes more important (Flood et al. 2025). In this case, the loss of these species may be partly driven by lemming herbivory, suggesting a shift in the food web may be as important as changes in competition.

Although our nutrient addition treatments have shifted the plant growth form and species composition towards those of fox dens, the plots are not, as of yet, complete mimics of fox den vegetation. This suggests it may take decades for the vegetation on a fox den or other areas of nutrient enrichment to reach a stable state. The largest difference between our experimental treatments and fox dens is a lack of tall shrubs. The species of tall shrubs found on fox dens are rare outside the den habitat, which raises the possibility that seeds may rarely find their way to a nutrient enriched spot. How fox dens have retained tall willow cover is not entirely clear. We suspect that tall willows first need a grass cover to build before willows invade a nutrient rich location. Our past work showed that the grass cover helped to explain increased snow depth on fox dens, but the effect of tall willows was twice as strong (Gharajehdaghipour and Roth 2018). We had assumed from the species composition we have seen on fox dens in the past that increased snow depth would allow the colonization by plants needing protection from the winter conditions. For instance, fox dens have two indicator species, *Rubus acaulis* and *Pyrola grandiflora.* These species are generally absent from the tundra heath habitat but are common in the boreal forest 30 km inland from our study site. These species likely benefit from the increased snow depth on the dens. It has also been suggested that plants’ response to fertilizer in tundra heath is limited by the thin snow cover, preventing the buildup of plants (Gough et al. 2002). However, changes in the plant community on our experimental plots were almost entirely the result of increased nutrients, not snow depth. As with tall willows, the colonization of suitable habitats by species requiring deep snow is quite unpredictable due to limited seed supply. This dispersal limitation would also explain the highly variable plant species composition found on den sites.

Our nutrient additions appear to mimic the nutrient additions on fox dens. The N applied to plots was not detectable in the soil by the following year, but P pools were. This pattern confirms our earlier work showing increased N content of plants on fox dens, no differences in soil inorganic N levels and higher levels of extractable P compared to reference areas (Gharajehdaghipour et al. 2016). The ubiquitous low inorganic N levels are consistent with the depletion of limiting soil nutrients after tundra green-up (Chapin et al. 1986; Rasmussen et al. 2021). The differences found in phytoavailable P are also consistent with recent plant uptake. No P was accessible by root interception, the easiest and most direct method of uptake (Bucher 2007), and was detected in most non-fertilized experimental plot samples. Our addition of sodium triphosphate in the fertilized plots should initially dissolve into this soil pool but can be readily accessed by organisms or chemically bound to other soil constituents (Bucher 2007), and we cannot rule out leaching in these sandy soils. The triphosphate added in the fertilizer treatment explains the different extractable soil P content between treatments when using citric and hydrochloric acid extractants; both methods detect weakly bound phosphates. The similar extractable soil P content of fertilized plots and fox dens relative to controls found using these two extractants and the null finding of the phosphatase extraction support long term inorganic phosphate deposition by foxes as the mechanism behind the elevated extractable P levels found on fox dens by Gharajhedaghipour et al. (2016). Overall, these trends show that N is the main limiting nutrient on tundra heaths, and denning activity provides relatively more P than N to meet the demands for plant growth. Working a dry heath site similar to ours in species composition, Gough et al. (2002) found a greater increase in grass growth in N and P, compared to just N fertilized treatments, suggesting an N and P colimitation. We did not find any effect of the treatments on soil moisture. We expected the extended buildup of organic soils from released production to increase retention of soil moisture in fertilized plots relative to controls, but it is either too early in the experiment for such an effect to develop or conditions at the time of sampling may have reduced detection.

The increases in moss and prostrate shrub cover observed in all vegetation plots suggest changing climate conditions over the last few years of this study. Data from the nearest weather station shows that from 2018 to 2024, the mean daily temperature during the growing season (June 1 to September 30) increased by 0.5 ˚C per year (Supplementary Fig. 5). The annual period of sea ice conditions that drive the arctic terrestrial dynamics in Western Hudson Bay is decreasing (Gupta et al. 2022), allowing for warmer and longer plant growth and decomposition periods. This climate change is expected to result in greater nutrient availability (Keuper et al. 2012) and growth of the existing prostrate shrub dominated community. Expansion of *D. integrifolia* cover, the dominant prostrate shrub in the heath habitat, may benefit collared lemmings, which rely on these communities for winter forage and habitat (von Beckerath et al. 2022). Should this effect extend across the Low Arctic, it could reverse decreasing lemming populations (Ehrich et al. 2020) and so indirectly benefit Arctic foxes and affect their alternative prey species (Johnson-Bice et al. 2025a).

Except for caribou, consumers generally increased their presence in response to fertilizer but not the deeper snow treatments. Over half of the insect OTUs were in the dipteran order and are therefore likely using the increased vegetation on fertilized plots as a habitat, not a food source. Studies in other heath vegetation have shown increased insect abundance and diversity with increased vegetation height (Haysom and Coulson 1998). For vertebrates, our results are generally similar to those of Zhao et al. (2022), who documented increased wildlife at den sites, including caribou, but not geese, which are common prey of foxes (McDonald et al. 2017). Canada geese preferred fertilized plots, so the failure of geese to increase activity at dens over reference sites in the earlier study may be due to their higher perceived predation risk associated with dens (Johnson-Bice et al. 2025b). In 2021, the only year with no difference in Canada goose use of fertilized and non-fertilized plots, their visitation to all plots was low. This year also had the lowest hatch success rate of Canada geese in a decade of monitoring nesting in the region, with 80% of nests lost to predation, likely due to a delay in nest initiation because of persistent snow cover and increased fox reproduction driven by increased lemming density (Johnson-Bice et al. 2025a). Lemmings were more active on fertilized plots in the summer, regardless of snow fencing but showed a preference for fertilized and snow fenced plots in the winter. These results support the hypothesis that lemmings are attracted to the thicker snow and higher-quality vegetation on Arctic fox dens posited by Gharajehdaghipour and Roth (2018). Lemming latrine and their associated nests have been shown to increase soil nutrient availability and microbial activity (Roy et al. 2022). The large number of winter latrines and goose feces could increase nutrient cycling, helping to maintain high nutrient availability in nutrient rich locations.

It has been suggested that the mammoth steppe biome that existed prior to the Holocene was maintained by a large density of herbivores, whose activities reduced low productivity vegetation and maintained elevated levels of soil nutrients, promoting vegetation that was more productive than the present tundra community (Zimov et al. 2012). Our study suggests that in tundra heaths, the concentration of nutrients at den sites could lead to a similar phenomenon where high nutrient vegetation attracts herbivores, which promotes nutrient cycling, creating a positive feedback between vegetation and soil.

## Supplementary Information

Supplementary material 1.

## Data Availability

The datasets used in this study and the R code used to analyze the data are available from the Borealis: 10.5683/SP3/PON3VU

## Abbreviations

GAM: Generalized additive model
glm: General linear model
OTU: Operational taxonomic unit
NMDS: Non-metric multidimensional scaling
