# Supplementary material for: Ecosystem engineering of tundra heath by Arctic fox (Vulpes lagopus) is driven by nutrient additions
Source: Ecol Process. 2025 Sep 23;14(1):80. doi: 10.1186/s13717-025-00646-5 (PMC12457229; doi:10.1186/s13717-025-00646-5)
Supplement: Supplementary file 1 — Supplementary material 1. [file 13717_2025_646_MOESM1_ESM.docx]

**Supplementary Table 1**. Net species colonization or loss from permanent quadrats from 2017 (pretreatment) to 2024 in each treatment. Values are the number of times (out of 20 permanent quadrats per treatment), there was an increase or decrease in a species occurrence in the repeatedly sample quadrats.

|  | Control | Fence | Fertilizer | Fence + fertilizer |
| --- | --- | --- | --- | --- |
| *Andronmeda polifolia* | 1 | 1 | 1 | 2 |
| *Arctostaphylos rubra* | -1 | 1 | 1 | -1 |
| *Bartsia alpina* | 1 | 0 | -1 | 0 |
| *Betula glandulosa* | 0 | 1 | 0 | 0 |
| *Carex rupestris* | -1 | 0 | -1 | 0 |
| *Cerastium alpinum* | 0 | 0 | 0 | 0 |
| *Chamaenerion angustifolium* | 0 | 0 | 1 | 6 |
| *Dryas integrifolia* | 0 | 0 | 0 | 0 |
| *Elymus mollis* | 1 | -1 | 5 | 9 |
| *Empetrum nigrum* | 0 | -1 | 0 | 0 |
| *Juncus albescens* | 0 | 0 | 0 | 0 |
| *Lesquerella arctica* | 3 | 5 | -3 | 2 |
| *Minuartia rubella* | 0 | 1 | 5 | 5 |
| *Moss sp* | 0 | 0 | 0 | 0 |
| *Oxytropis campestris* | 1 | 0 | -1 | 0 |
| *Pedicularis flammea* | 1 | -1 | 0 | 0 |
| *Pinguicula vulgaris* | 2 | 0 | 0 | 0 |
| *Pleurozium* | 0 | 0 | 3 | 1 |
| *Poa alpigena* | 1 | 0 | 0 | 2 |
| *Polygonum vivparaum* | 1 | 1 | 0 | 0 |
| *Pyrola grandiflora* | 0 | 0 | 0 | 0 |
| *Rhododendron lapponicum* | 1 | 1 | 3 | -11 |
| *Salix lanata* | 1 | 0 | 0 | 2 |
| *Salix planifolia* | 0 | 0 | 0 | 1 |
| *Salix reticulata* | 0 | 0 | 0 | 4 |
| *Saxifraga aizoides* | 5 | 1 | -6 | -5 |
| *Saxifraga oppositifolia* | -4 | 4 | -5 | 1 |
| *Saxifraga tricuspidata* | -1 | 0 | 7 | 2 |
| *Scirpus caepetosis* | 0 | 3 | 0 | 1 |
| *Shepherdia canadensis* | 0 | 0 | -1 | 0 |
| *Stellaria longipes* | 0 | 0 | 4 | 1 |
| *Tofeldia pusilla* | 0 | 2 | -1 | -8 |
| *Vaccinium uligonisum* | 1 | 4 | -2 | 1 |
| *Vaccinium vitis idea* | 0 | -1 | 0 | 0 |

**Supplementary Table 2.** Total number of operational taxonomic units (OTUs) assigned to each arthropod order. Order OTU totals for vegetation plot treatment groups and fox dens are included for cross comparison. One Araneae is included here but was not used in the analysis.


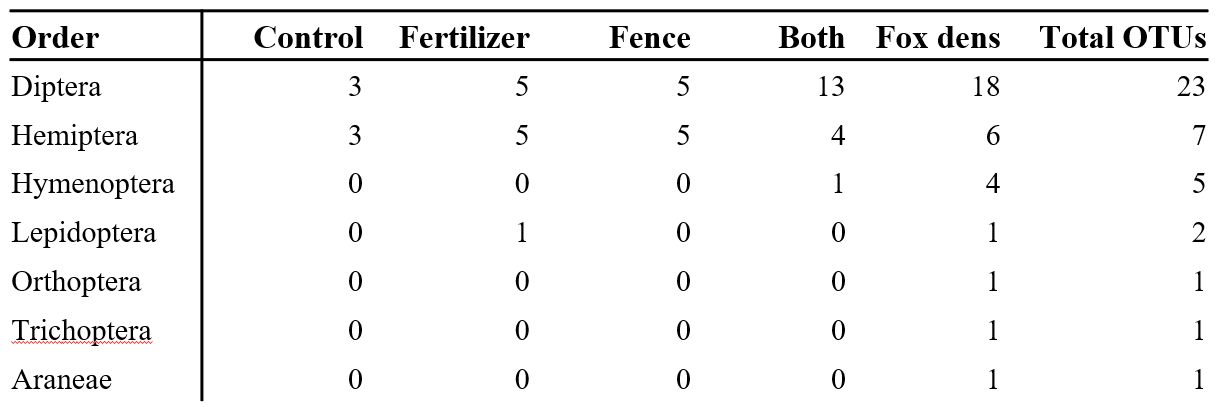


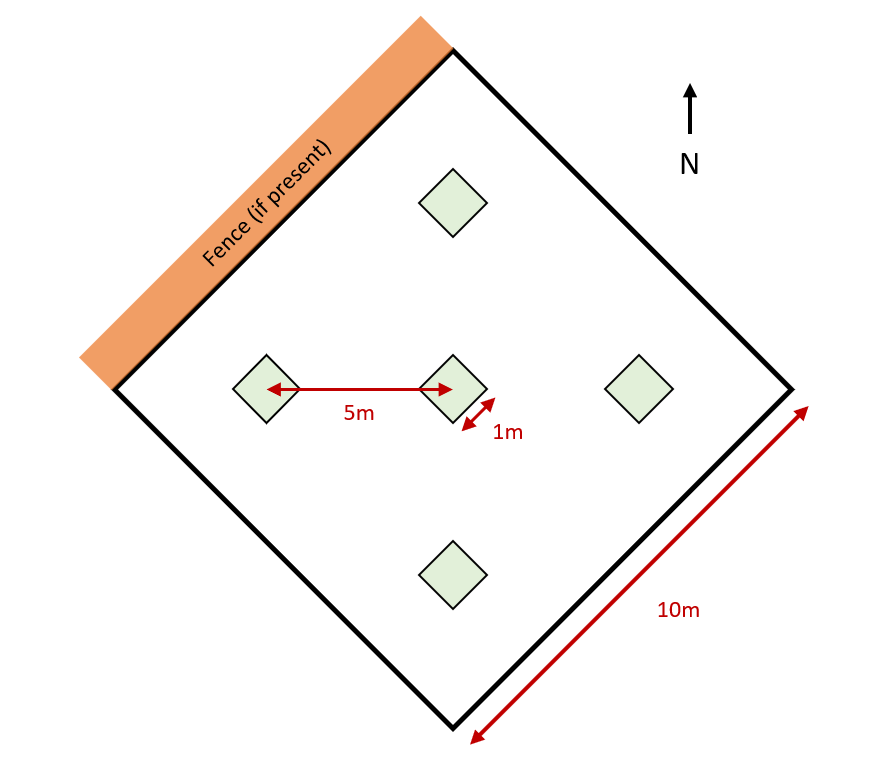


**Supplementary Figure 1.** Configuration of experimental plots. Permanent 1m^2^ quadrats (in green) were used to measure plant species cover in late July to early August each year. On half the plots fencing was set up on the windward NW edge.


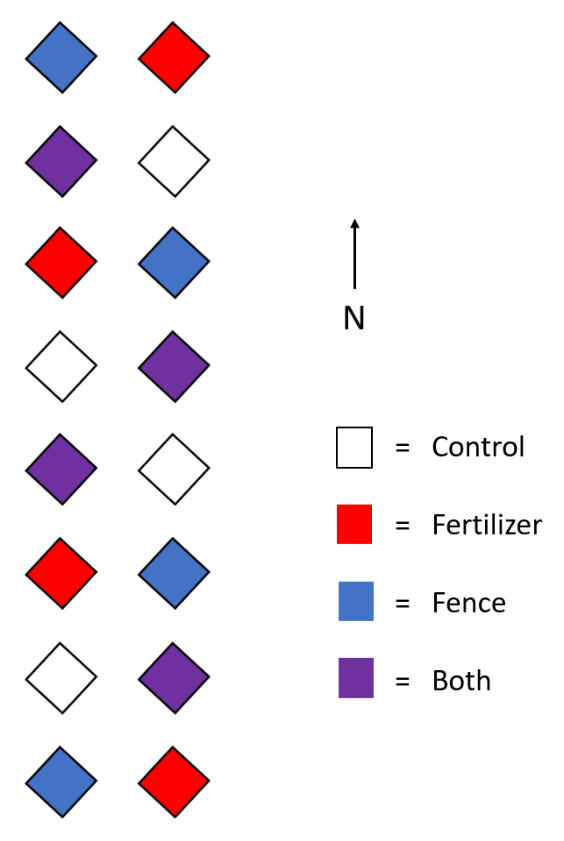


**Supplementary Figure 2.** Spatial arrangement of experimental plot.


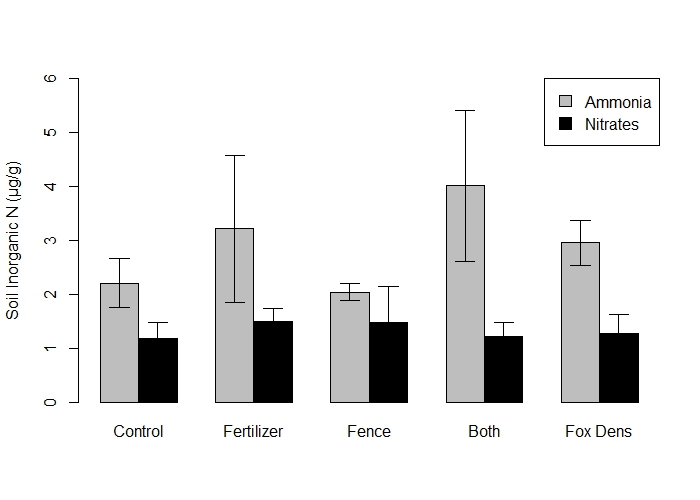


**Supplementary Figure 3.** Inorganic N content of experimental plot and fox den soil samples collected in July 2022. Experimental plot treatment groups (Control, Fertilizer, Fence, Both) show means of 4 sites each and the fox den group shows means of 8 sites. Error bars represent standard error.

**
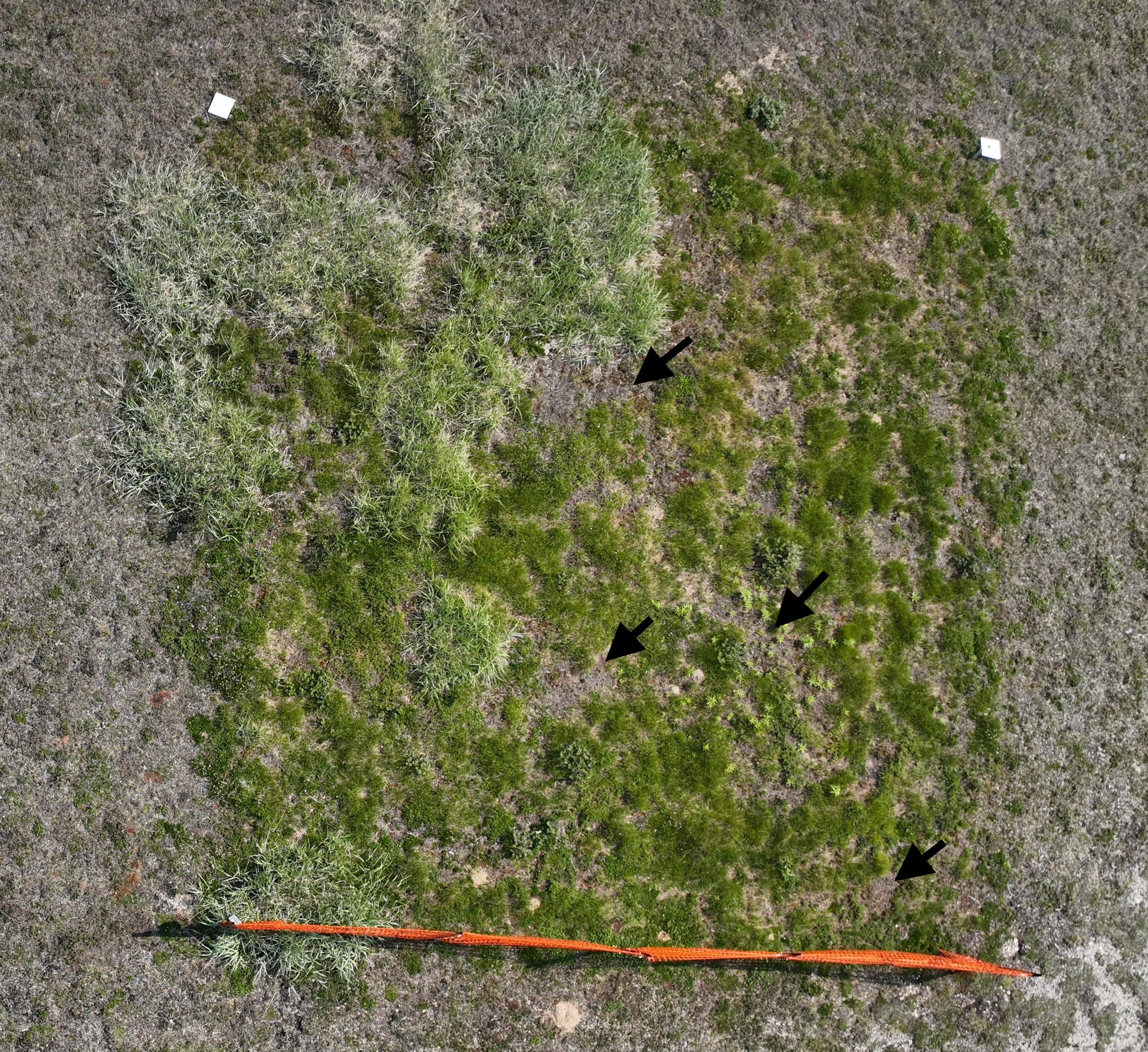
**

**Supplementary Figure 4.** Patches of dead *Dryas integrifolia* (black arrows) in a fertilizer and snow fence plot with a large number of winter lemming latrines. Elymus molis dominates the upper left region of the plot.

**Supplementary Figure 5.** Mean daily temperature from 2018 to 2024 for the period from June 1 to September 30. Data from the Churchill weather station (ID 5060605). Available from: https://climate.weather.gc.ca/historical_data/search_historic_data_e.html
